# Prevalence and self-reported reasons of cannabis use for medical purposes in USA and Canada

**DOI:** 10.1007/s00213-021-06047-8

**Published:** 2022-01-12

**Authors:** Janni Leung, Gary Chan, Daniel Stjepanović, Jack Yiu Chak Chung, Wayne Hall, David Hammond

**Affiliations:** 1 National Centre for Youth Substance Use Research, The University of Queensland, Brisbane, Australia; 2 School of Psychology, The University of Queensland, Brisbane, Australia; 3 School of Public Health and Health Systems, University of Waterloo, Waterloo, Canada

**Keywords:** Cannabis, Marijuana, Therapeutic use, Prevalence, Epidemiology, Legalisation

## Abstract

**Rationale:**

There has been increasing attention on cannabis use for medical purposes, but there is currently a lack of data on its epidemiology.

**Objectives:**

To examine the epidemiology of self-reported cannabis use for medical purposes by (1) estimating its prevalence, (2) comparing gender and age differences, and (3) investigating what reasons they were used to manage.

**Methods:**

Participants included 27,169 respondents (aged 16–65) who completed Wave 1 of The International Cannabis Policy Study (ICPS) conducted across Canada and the USA in 2018 via online surveys. Cannabis policy conditions were “US legal–recreational” (legal for both recreational and medical uses), “US legal–medical only”, “US illegal”, and “Canada–medical only”.

**Results:**

The overall prevalence of self-reported ever cannabis use for medical purposes was 27%, with similar rates by sex and the highest prevalence in young adults. Prevalence was higher in US legal–recreational states (34%) than US illegal states (23%), US legal–medical only states (25%), and Canada (25%). The most common physical health reasons include use to manage pain (53%), sleep (46%), headaches/migraines (35%), appetite (22%), and nausea/vomiting (21%). For mental health reasons, the most common were for anxiety (52%), depression (40%), and PTSD/trauma (17%). There were 11% who reported using cannabis for managing other drug or alcohol use and 4% for psychosis.

**Conclusions:**

A substantial proportion of the North American population self-reported cannabis use for medical purposes for a variety of medical reasons, including those living in jurisdictions without legal markets. Further research is needed to understand the safety and efficacy of these forms of medical cannabis use.

**Supplementary Information:**

The online version contains supplementary material available at 10.1007/s00213-021-06047-8.

## Introduction

Medical cannabis refers to the prescription of cannabis or cannabinoids to alleviate symptoms of a medical condition or to treat a disease. The two major cannabinoids found to possess therapeutic properties are cannabidiol (CBD) and Δ^9^-tetrahydrocannabinol (THC). THC induces psychoactive and anxiogenic effects, while CBD is reported to be an anxiolytic and to modulate the psychoactive effects of THC. Medical cannabis can be administered by multiple routes, e.g. as cannabis edibles, vaporizable and smokable cannabis, oils, and capsules. Reviews and meta-analyses have found some evidence to support the use of medical cannabis to treat nausea and vomiting during chemotherapy, chronic pain, and epilepsy (Jensen et al. 2015; Whiting et al. 2015; Wong & Wilens, 2017).

North America includes some of the first jurisdictions to allow cannabis use for medical purposes. Regulated medical cannabis use became legal in Canada in 2001 (Rough, 2017), regulated under the “Access to Cannabis for Medical Purposes Regulations” from 2014 (Government of Canada, 2020). Healthcare practitioners can authorize cannabis for patients to relieve listed refractory symptoms, such as severe nausea and vomiting from chemotherapy, insomnia, and depressed mood associated with chronic diseases and symptoms encountered in palliative care. As of writing, 36 states in the USA allow cannabis use for medical purposes. The Food and Drug Administration has approved four cannabinoid drugs for prescription use, namely, cannabidiol (Epidiolex), dronabinol (Marinol), CX dronabinol (Syndros), and nabilone (Cesamet) (U.S. FDA, 2020). Medical conditions approved for cannabis use vary among states, where most states allow patients suffering from epilepsy, cancer, and multiple sclerosis to be have medical cannabis prescribed or recommended (National Conference of State Legislatures, 2020). Yet, for most types of medical use, there is no recommended dosage for medical cannabis products and weakly regulated medical cannabis programmes in Canada and some US states have created a blurry boundary between medical and non-medical uses (Hall et al. 2019).

Some of the conditions approved for medical use in Canada and certain US states lack evidence on their effectiveness and safety (Hall et al. 2019). There are insufficient studies to draw strong conclusions on the efficacy of medical cannabis for many of these uses, as most studies are often based on case reports, relied on small self-selected patient cohorts, or lack a randomized control comparison condition (Victorian Law Reform Commission, 2015). A systematic review found that a majority of studies on the therapeutic effects of cannabis in treating anxiety symptoms were classified as low in quality of evidence, where symptoms of mental disorders did not significantly improve from pharmaceutical THC use (with or without CBD), but instead adverse effects were experienced by a substantial number of people (Black et al. 2019).

Despite federal prohibition of cannabis use in the USA, people may engage in self-medication, defined as use of cannabis for medical reasons without authorization, rather than authorized or prescribed use. Self-medication of cannabis has long been common to use for chronic conditions and mental health symptoms (Bouso et al. 2020; Sarvet et al. 2018). Using substances by self-medication may increase the risks of adverse effects, incorrect manner of administration, incorrect dosage, dangerous drug interactions, and risk of dependence and abuse (Ruiz, 2010). For example, self-medication of cannabis to treat inflammatory bowel disease (IBD) symptoms was associated with a higher risk of developing depression symptoms and an increased vulnerability to substance misuse compared with using cannabis recreationally (Hansen et al. 2020).

In recent years, as more countries have moved towards cannabis legalization, there have been increased efforts by practitioners, researchers, and policymakers to evaluate the efficacy and adverse effects of using cannabis for medical purposes. There are concerns that recreational and medical cannabis legalization may increase the prevalence of use and cannabis use disorders among youth (Hansen et al. 2020; Leung et al. 2019). Nevertheless, previous findings indicated that states with legalized medical cannabis laws have a higher prevalence of cannabis use for self-medication purposes on mood and anxiety disorders (Sarvet et al. 2018). In addition, most medical cannabis users also use cannabis recreationally (Wall et al. 2019).

It is important to gain a better understanding of the prevalence of medical cannabis use and its relationship to the jurisdictional legality of medical cannabis use and the main reasons for its use. There is inadequate representative data on the prevalence of medical cannabis use, the reasons for its use, and the characteristics of medical cannabis users. There are also limited comparisons of medical cannabis use in US states with different recreational and medical cannabis laws, as most literature on the prevalence of medical cannabis users has focused on specific patient groups or had relatively small samples (Victorian Law Reform Commission, 2015).

This study aims to examine the epidemiology of self-reported cannabis use for medical purposes by (1) estimating the self-reported prevalence of cannabis use for medical purposes in North America in jurisdictions that vary in their recreational and medical cannabis laws, (2) comparing the prevalence of cannabis use as by gender and age groups, and (3) investigating what physical or mental conditions medical cannabis was self-reported to manage.

## Methods

### Survey design

The International Cannabis Policy Study (ICPS) examines five primary research questions, including the extent to which legalization is associated with changes in (1) prevalence, consumption, and patterns of cannabis use; (2) commercial retail environment, price, and purchasing; (3) risk behaviours, including driving after cannabis use and use in “high-risk” occupational settings; (4) perceptions of risk and social norms; and (5) effectiveness of specific regulatory policies, including advertising restrictions, product labelling and warnings, public education campaigns, and the use of cannabis in public spaces (Hammond D, 2018). It is conducted in Canada and the USA and consists of population-based surveys conducted annually since 2018, immediately prior to non-medical legalization in Canada. This study draws on data from Wave 1. These were collected via self-completed web-based surveys conducted from August 27 to October 7, 2018, with respondents aged 16–65.

Respondents were recruited through the Nielsen Consumer Insights Global Panel and their partners’ panels. The Nielsen Panels are recruited using probability- and non-probability-based sampling methods. Quotas are used for recruitment in Canadian states and provinces. In addition, post-stratification weights reflect age, sex, and race/ethnicity distribution within provinces and states. All population-based samples have some level of bias; the publicly availability Technical Report for each survey wave provides a detailed breakdown of the sample profile, including comparisons with population distribution for both sociodemographic features and cannabis use indicators (see http://cannabisproject.ca/methods/). Overall, the ICPS samples match sociodemographic distributions in Canada and the USA. Prevalence of cannabis use is moderately higher among the ICPS sample compared to state-level estimates from NSDUH; however, this reflects that the ICPS sample does not include respondents above 65 years old.

Email invitations (with a unique link) were sent to a random sample of panellists (after targeting for age [16–65] and country [USA or Canada] criteria); panellists who were ineligible (not aged 16–65 or not living in the USA or Canada) were not invited. Surveys were conducted in English in the USA and English or French in Canada. The median survey time was 19.9 min.

### Consent and ethics

Respondents provided consent prior to completing the survey. Respondents received remuneration in accordance with their panel’s usual incentive structure (e.g. points-based or monetary rewards, chances to win prizes). The study was reviewed by and received ethics clearance from the University of Waterloo Research Ethics Committee (ORE# 22,392/31330). A full description of the study methods can be found in the International Cannabis Policy Study: Technical Report – Wave 1 (2018) (Hammond et al. 2018).

### Participants

A total of 28,471 respondents aged 16–65 (mean = 45.5, SD = 15.5) completed the survey; 1302 participants were removed due to invalid responses to data quality questions, ineligible country of residence, smartphone use, or residence in District of Columbia (inadequate sample size). The analytic sample consisted of 27,169 participants, of whom 58% responded that they have ever tried cannabis (Hammond et al. 2018). In the survey, participants were discouraged from attempting to complete the main survey via a mobile device, but were not restricted from doing so. However, the smaller screen size of smartphones can alter the way online surveys are rendered in ways that require greater “scrolling” and smaller rendering of images in ways that may degrade data quality; therefore, responses completed via smartphones were excluded to avoid this potential bias.

### Sample weights

Post-stratification sample weights were constructed based on the Canadian and US Census estimates to generate results that are applicable to the population. Respondents from Canada were classified into age-by-sex-by-province and education groups. Respondents from the US states where cannabis was legal for recreational use were classified into age-by-sex-by-legal state, education, and region-by-race groups, while those from the states where cannabis was illegal were classified into age-by-sex, education, and region-by-race groups. Correspondingly, grouped population count and proportion estimates were obtained from Statistics Canada (Statistics Canada, 2016, 2017) and the US Census Bureau (U.S. Census Bureau, 2018a, 2018b). A raking algorithm was applied to the full analytic sample (*n* = 27,169) to compute weights that were calibrated to these groupings. Weights were rescaled to the sample size by states/provinces and countries. Estimates are weighted unless otherwise specified. Sample sizes for the states are reported in the ICPS technical report available at http://cannabisproject.ca/methods/ (Supplementary material S7). The purpose of weighting is to ensure similar distribution of the sample with known population parameters. This is particularly important given that cannabis use is known to vary across sociodemographic subgroups, such as by sex at birth. Otherwise, discrepancies between the sample and population on these sociodemographic variables can confound the point estimates. It is entirely appropriate to weight both probability-based and non-probability-based samples to enhance prevalence estimates in this way.

### Measures

**Fig. 1:**
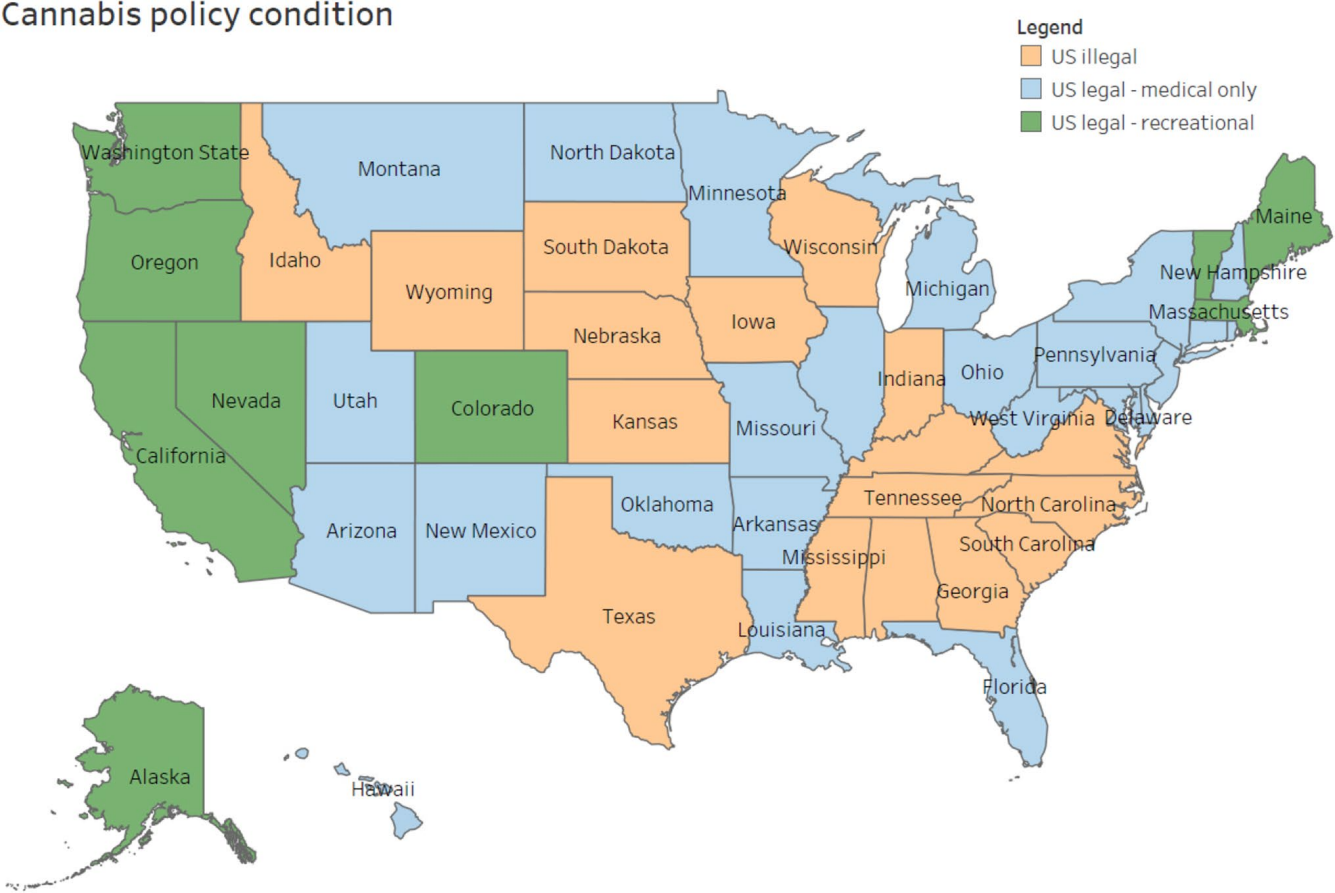
Map of cannabis policy conditions at the time of the survey in 2018: “US–illegal”, illegal for recreational use and no legal medical market; “US legal–recreational”, legal for both recreational and medical; “US legal–medical only”, legal medical market, but illegal for recreational use

Cannabis policy conditions in the participants’ jurisdiction of residence were categorized in terms of recreational and medical cannabis laws. At the time of the survey in 2018, cannabis was legal for medical use only in Canada (illegal for recreational use). In the USA, all the states that had legalized recreational cannabis had also legalized medical cannabis. Therefore, the three US policy conditions operationalized were (1) “US legal–recreational”, legal for both recreational and medical; (2) “US legal–medical only”, legal medical market, but illegal for recreational use; (3) “US–illegal”, illegal for recreational use and no legal medical market (see Fig. 1 and Supplementary material S1).

Self-reported reasons for medical cannabis use were measured by a set of items that asked participants, “Have you ever used marijuana to improve or manage symptoms for any of the following:” The physical conditions included headaches/migraines, pain (including arthritis, neuropathy, or menstrual cramps/PMS), nausea/vomiting (including managing chemotherapy), lack of appetite, seizures, muscle spasms, to treat cancer/tumours, problems sleeping, or others. Mental health reasons included anxiety (including phobia, obsessive–compulsive disorder, or panic disorder), depression (including dysthymia), post-traumatic stress disorder (PTSD) or traumatic event (e.g. abuse or loss), bipolar disorder or mania, psychosis (e.g. paranoia, disorganized thinking, hearing voices that others cannot hear), schizophrenia, drug or alcohol use, or “other”. Participants who responded “yes” for any of the conditions or reported any other reasons were coded as a prevalent case for self-reported cannabis use for medical purposes.

Gender was measured by asking, “How would you describe your gender identity today?” Responses were female, male, other, or unstated. There was a very low proportion of people who selected other or had unstated responses (< 1%), and so cell sizes were too small for analysis. These were recoded as missing, then redistributed using multiple imputations based on all used variables in our analyses to minimize potential bias. Control variables included ethnicity (white/other) and education (less than high school/high school diploma or equivalent/ some college or technical or vocational training or certificate or diploma, or apprenticeship, or some university/bachelor degree or higher).

### Data analysis

Missing data were imputed using multiple imputations with five iterations for the 1% who have ever used cannabis and did not report that they have never used cannabis for any of the medical reasons, but did not answer or responded “don’t know” for the medical reason of cannabis use items (physical reasons *n* missing = 274; mental health reasons *n* missing = 335). Estimates presented are pooled from the imputed datasets.

Weighted prevalence of self-reported cannabis use for medical purposes was estimated in jurisdictions that vary in cannabis policy conditions (detailed above) and compared by chi-squared tests. The whole sample was used as the denominator, including those who had not tried cannabis included as non-cases. Prevalence estimates were compared by age and gender groups.

Multiple logistic regression analyses were conducted to examine the associations between medical cannabis use for any reason by age, gender, and cannabis policy conditions (“US illegal” as reference), controlling for education and ethnicity. Odds ratios and 95% confidence intervals (CI) were estimated. Among participants who have self-reported cannabis use for medical purposes, frequencies by each of the medical condition and mental health reasons were presented to investigate what the participants used cannabis to manage. The odds ratios of use for each of the reasons were analysed in separate multiple logistic regression models. All models included cannabis policy conditions, age, sex, and controlled for education and ethnicity. Analyses were conducted in R using StatsNotebook (Chan, G., StatsNotebook Team, 2020).

## Results

### Prevalence of self-reported cannabis use for medical purposes

Across Canada and the USA, 27.1% (26.6–27.6%) of participants self-reported ever cannabis use for medical purposes. Between the cannabis policy conditions, the prevalence was significantly higher in US legal states (34.0% (32.9–35.1%), *p* < 0.001) than in US illegal states (22.8% (21.4–24.2%)), in US medical only states 25.4% (24.3–26.5%), and in Canada (medical only; 24.7% (23.8–25.5%); see Table 1).

**Table 1:** Prevalence of self-reported ever cannabis use for medical purposes by conditions of jurisdiction

	Sample size	Unweighted % (95% CI)	Weighted % (95% CI)
Across all jurisdictions	27,169	24.5% (23.9–25.0%)	27.1% (26.5–27.7%)
Cannabis policy condition***^,†^			
Canada–medical only	10,057	22.4% (21.6–23.2%)	24.7% (23.8–25.5%)
US legal–recreational	7398	31.8% (30.7–32.9%)	34.0% (32.9–35.1%)
US legal–medical only	6018	22.0% (20.9–23.0%)	25.4% (24.3–26.5%)
US illegal	3696	19.7% (18.4–21.0%)	22.8% (21.4–24.2%)

^***^Chi-squared tests showed *p* < 0.001

^†^ “US illegal”, illegal for recreational use and no legal medical market; “US legal–recreational”, legal for both recreational and medical

**Fig. 2:**
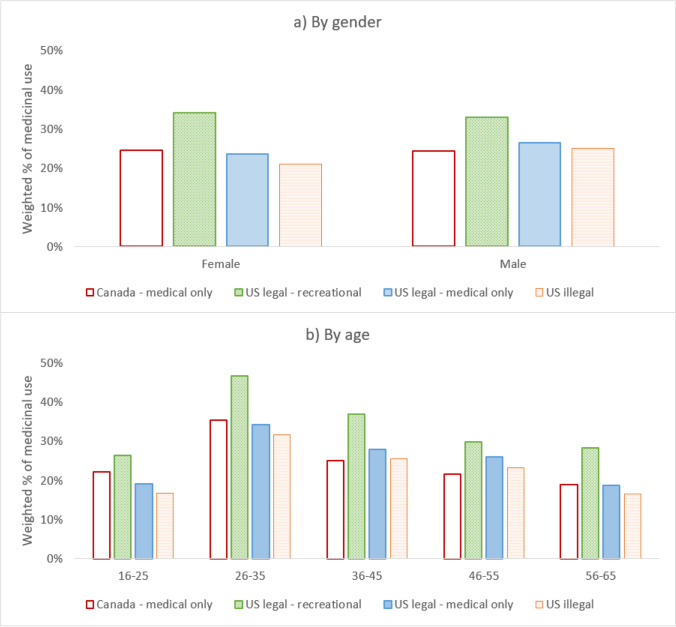
Prevalence of self-reported ever cannabis use for medical purposes by cannabis policy condition by **a** gender and **b** age groups (see Supplementary material S2 for data tables)

Patterns of self-reported cannabis use for medical purposes by the policy conditions were in general consistent across gender and age groups. Prevalence was highest in young adults aged 26–35 in US legal–recreational states (see Fig. 2; data tables are available in Supplementary material S2).

### Odds of cannabis use for medical purposes by policy condition, gender, and age

Compared to people who lived in US illegal states, odds of self-reported cannabis use for medical purposes were 1.75 (95% CI = 1.59,1.93) higher in those who lived in US legal–recreational states. People who lived in US legal–medical only states (OR = 1.17 [1.06,1.29]) and Canada (OR = 1.11 [1.01,1.22]) also had higher odds of self-reported cannabis use for medical purposes. Males had higher odds of self-reported cannabis use for medical purposes (OR = 1.09 [1.03,1.15]) than females. Young people aged 26–35 had 2.16 (95% CI = 1.95,2.39) higher odds of self-reported cannabis use for medical purposes (see Table 2).

**Table 2:** Logistic regression on self-reported ever cannabis use for medical purposes

	Adjusted odds ratios			
	OR	95% CI		***p***
		Lower	Upper	
Gender				
Female	1.00	(Ref)		
Male	1.09	1.03	1.15	0.002
Age groups				
16–25	1.00	(Ref)		
26–35	2.16	1.95	2.39	< 0.001
36–45	1.39	1.24	1.55	< 0.001
46–55	1.06	0.95	1.18	0.274
56–65	0.81	0.72	0.90	< 0.001
Cannabis policy condition				
Canada–medical only	1.11	1.01	1.22	0.028
US legal–recreational	1.75	1.59	1.93	< 0.001
US legal–medical only	1.17	1.06	1.29	0.003
US illegal	1.00	(Ref)		

Model of gender, age, and policy condition on cannabis use for medical purposes, controlling for education and ethnicity (see Supplementary material S6 for data on control variables)

### Physical or mental health reasons for use

**Fig. 3:**
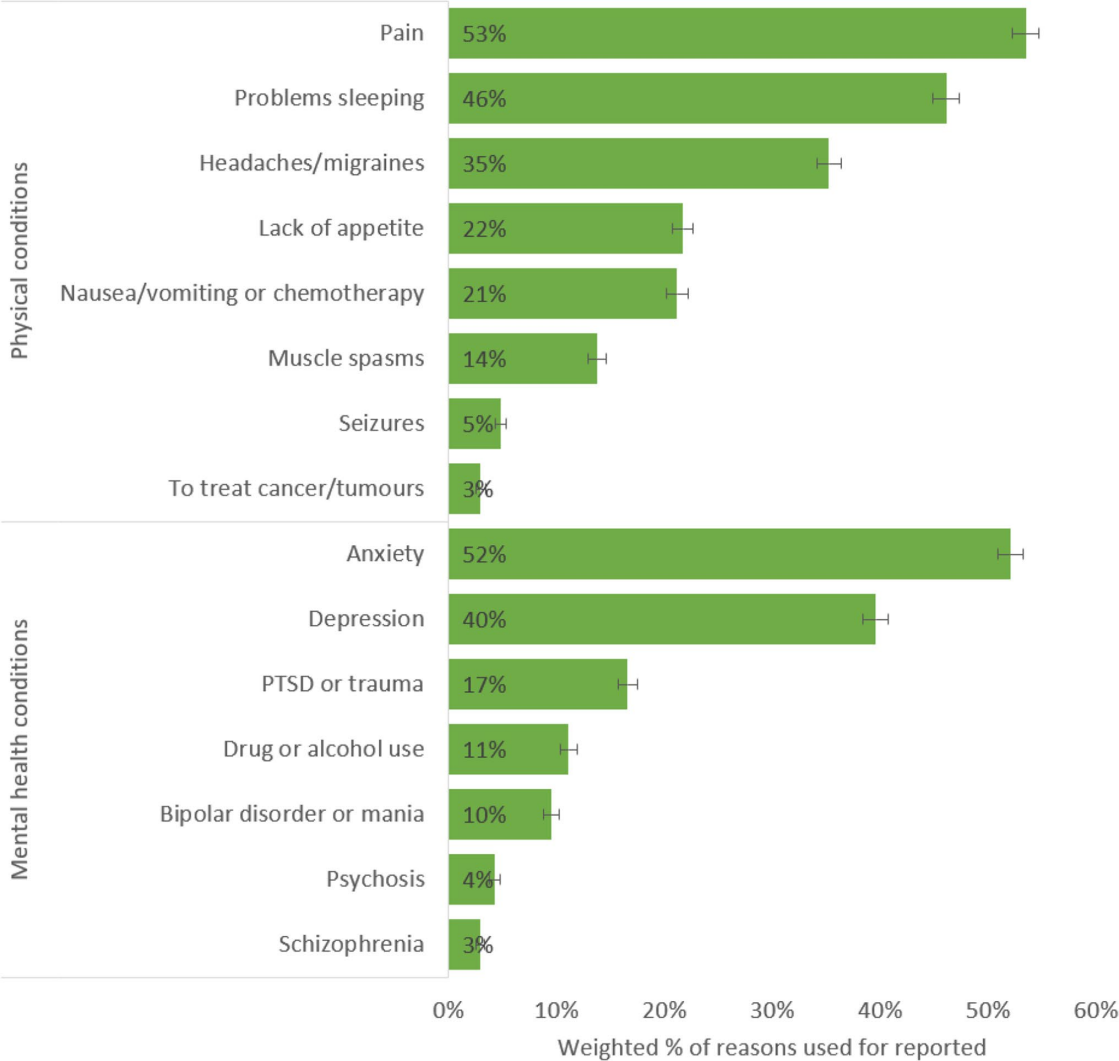
Weighted prevalence of reasons for which cannabis was ever used to manage, among people who self-reported cannabis use for medical purposes (see Supplementary material S3 for data tables)

Among those who had self-reported the use of cannabis for medical purposes, the most commonly reported physical reasons were to manage pain (53%), problems sleeping (46%), headaches or migraines (35%), lack of appetite (22%), and nausea or vomiting (21%; see Fig. 3; data table available in Supplementary material S3).

For self-reported mental health reasons, the most common uses were reported for anxiety (52%), depression (40%), post-traumatic stress disorder (PTSD), or trauma (17%; see Fig. 3; data table available in Supplementary material S3). There were 11% who reported using cannabis to manage other drug or alcohol use, 4% to manage psychosis, and 3% to manage symptoms of schizophrenia.

There were interrelationships between common self-reported physical and mental health reasons for cannabis use. Cannabis use for managing anxiety, depression, pain, headaches or migraines, and problems sleeping were intercorrelated (*r* = 0.45–0.61, *p* < 0.001; see Supplementary material S4). Self-reported use to manage symptoms of PTSD or trauma was associated with use for anxiety or depression (*r* = 0.38, *p* < 0.001). Use for nausea/vomiting was associated with use for lack of appetite (*r* = 0.46, *p* < 0.001).

Multiple logistic regression models showed that females were more likely to use cannabis for headaches or migraines, nausea or vomiting, problems sleeping, anxiety, and PTSD or trauma (see Supplementary material S5). Males were more likely to report cannabis use for the management of seizures, cancer or tumours, bipolar or mania, psychosis or schizophrenia, and other drug or alcohol use problems. Data for the control variables are available in Supplementary material S6.

## Discussion

The first US jurisdictions to legalize cannabis for medical use was California in 1996, and most Americans now reside in a state with legal access to medical cannabis. Despite this relatively long history, the demographic characteristics of medical cannabis users and the conditions that they seek to treat with cannabis have remained poorly understood. This paper sought to understand the epidemiology of self-reported cannabis use for medical purposes by utilizing the International Cannabis Policy Study, a population-based survey conducted with national samples of 16–65-year-olds in Canada and the USA.

To our knowledge, this is the first USA- and Canadian-wide study that estimated prevalence of self-reported cannabis use for medical purposes across jurisdictions of different recreational and medical cannabis use policies. Prevalence and forms of recreational cannabis use had been previously published (Goodman et. al. 2020a). Results indicated a higher prevalence of self-reported cannabis use for medical purposes in the USA than in Canada, noting that the survey was conducted immediately prior to legalization of cannabis for recreational use in Canada (legal for medical use only). Prevalence rates differed significantly as a function of the legal status of cannabis. One in three respondents in states that had legal recreational cannabis sales reported medical cannabis use. Conversely, states that did not allow legal recreational or medical cannabis access had the lowest rates of cannabis use with only just above one in five for respondents indicating that they used medical cannabis. Overall, the presence of a cannabis market, recreational or medical, was associated with a higher prevalence of self-reported cannabis use for medical purposes than the absence of a market. This is consistent with data from household surveys of US adults, which has found that all forms of cannabis use are highest in states with legal recreational markets, followed by those with legal medical markets, and lowest in states with no legal access to cannabis (Carliner et al. 2017; Obradovic, 2019). While this could be an effect of legalization, it also reflects higher prevalence of use prior to legalization in many cases.

The most common medical ailment for which participants reported using cannabis was the management of pain. This may reflect the analgesic properties of cannabinoids, which have been found to produce clinically significant reductions in pain in a minority of chronic pain patients, although the proportion is only marginally greater than that in patients who received a placebo (Stockings et al. 2018). A further factor influencing the use of cannabis for pain relief may be the growing interest in its use as a substitute for opioids, prompted by the epidemic of opioid overdose deaths in the USA (Guy et al. 2017). Individuals experiencing pain may seek out medical cannabis, and doctors may avoid prescribing opiates as a result of increased regulation and monitoring of prescriptions (Chang et al. 2018).

To manage or improve symptoms of anxiety was the most common reason for use among participants who reported using medical cannabis for mental health. Our finding was consistent with an online survey of medical cannabis users registered with a Canadian licensed producer, which found that anxiety was the most common mental health condition that cannabis was prescribed to treat (Turna et al. 2019). This could be a reflection of the anxiolytic properties of cannabinoids, especially the use of CBD, which is suggested as an adjunctive treatment for anxiety or stress-related disorders (Sharpe et al. 2020). The possible anxiolytic effects of CBD and other cannabinoids are demonstrated to mediate anxiety, stress, and restlessness in several animal and human studies (Crippa et al. 2009; Fraguas-Sánchez & Torres-Suárez, 2018). However, there is also a strong positive association between cannabis use disorder and anxiety disorder, because frequent cannabis users are more likely to have anxiety disorders, and anxiety disorder patients have a higher risk to develop cannabis dependence (Kedzior & Laeber, 2014). More evidence is needed on the effectiveness of cannabis in alleviating anxiety symptoms.

### Limitations

This study is subject to limitations common to survey research. Respondents were recruited using non-probability-based sampling, so the raw data do not provide nationally representative estimates. Therefore, the data were weighted by age group, sex, and region in both countries, and region-by-race in the USA. As explained in our methods, this adjusts our sample to have a similar distribution to the known population parameters. However, the study sample was somewhat more highly educated than the national population in the USA. In both countries, the ICPS sample had poorer self-reported general health compared to the national population, which is a feature of many non-probability samples (Fahimi et al. 2018). This may be partly due to the use of web surveys, which provide greater perceived anonymity than in-person or telephone-assisted interviews often used in national surveys (Hays et al. 2015). The rates of cannabis use were also somewhat higher than some national estimates, but this was likely because the ICPS sampled individuals aged 16–65 whereas the national surveys included older adults, who have lower rates of cannabis use than younger adults. The ICPS is also conducted online, whereas most national surveys are conducted in person. Compared to interviewer-assisted survey modes, self-administered surveys can reduce social desirability bias by providing greater anonymity for sensitive topics, including substance use (Dodou & de Winter, 2014; Kohut et al. 2012). The Canadian surveys were conducted in Canadian national languages of French and English. However, the US surveys were only conducted in English; therefore, the US sample may have under-represented people who did not speak English (e.g. Spanish speaking only). We excluded responses from smartphone use to avoid biases in data quality, as explained in the “Methods” section. However, our sample may have under-represented people without internet access outside of mobile devices. We did not have an adequate sample size of people who identified with non-binary gender, so our results may not be applicable in this minority population. Future research on the impacts of cannabis policy among ethnic minorities and disadvantaged groups are warranted.

Our study is a self-report survey of cannabis use for medical reasons. Individuals may self-rationalize or self-decided to use cannabis for medical reasons. This may explain why some of our participants reported using cannabis to help with some conditions that medical cannabis is not approved for, e.g. psychosis and schizophrenia. There is a distinction between authorized medical cannabis use under approval and supervisor by a health professional, compared to self-medication of cannabis for self-decided medical reasons. Consumers may define “medical use” broadly and the distinction between recreational and medical uses may be arbitrary in many cases, particularly with respect to relieving stress or enjoyment. We reported on self-reported common physical or mental health reasons for cannabis use for medical purposes. These reasons are not exhaustive. There were few rare participants who reported less common reasons; < 0.5% reported that they used medical cannabis for other reasons such as digestion, eating disorder, and attention problems. Future studies may review the list of conditions presented in the response options to ensure that common self-reported reasons for use are up-to-date. For future research, it would be important to examine whether cannabis users have received medical authorization for their self-reported cannabis use for medical purposes. This will help to illustrate the distinction between prescribed medical use, compared to a potentially large proportion of consumers who may be self-medicating for medical purposes without advice by a health professional. It would also be crucial to examine how people obtained medical cannabis in jurisdictions which did not allow legal recreational or medical cannabis access. This may allow us to differentiate risks and effects of medical cannabis obtained through legal medical prescription or other sources, so as to better inform policymakers and practitioners.

People who live in jurisdictions that have legalized cannabis use have higher exposure to cannabis marketing (Rup et al. 2020), and trends in reasons for cannabis use may be affected by promotional activities.

For example, cannabis is being promoted to reduce morning sickness and nausea to pregnant women, but cannabis use during pregnancy is associated with poorer birth outcomes (Hall et al. 2019). Goodman et al.’s (2020a, b) study on the use and perception of cannabidiol (CBD) products as part of the International Cannabis Policy Study shed light on CBD use in the USA and Canada. The marketing of CBD products and belief that CBD oil is beneficial for health are widespread, including for conditions for which there is little or no evidence of efficacy. Given the increasing ease of access to information, and misinformation, from online sources, it is important to generate evidence-based information on the benefits and risks of cannabis that are accessible to the general population. It is also important to conduct research into the uses of cannabis being promoted for by the industry and on social media to inform the communication of science-based information to the public.

### Conclusion

A substantial proportion of the North American population report ever using cannabis to improve or manage symptoms of medical or mental health conditions. This includes those who live in jurisdictions where there were no legal medical cannabis markets. Use is most common among young adults who would be expected to have lower rates of the chronic medical conditions for which medical cannabis is used for, such as chronic pain, than older adults. While there is emerging evidence of therapeutic effects of cannabis for certain conditions, there are some conditions that have no empirical support for, and others that cannabis can have adverse effects (e.g. psychosis). Our findings have implications for how cannabis use are being used by the population, which may included authorized use with prescription by a health professional, and also self-defined medical use without professional guidance. Further research on the epidemiology of medical cannabis use is needed to understand populations who may experience unintended negative health outcomes from their medical cannabis use.

## Supplementary Information

Below is the link to the electronic supplementary material.

Supplementary file1 (DOCX 278 KB)
