# Supplementary material for: Prevalence and self-reported reasons of cannabis use for medical purposes in USA and Canada
Source: Psychopharmacology (Berl). 2022 Jan 12;239(5):1509–19. doi: 10.1007/s00213-021-06047-8 (PMC9110511; doi:10.1007/s00213-021-06047-8)
Supplement: Supplementary file 1 — Supplementary file1 (DOCX 278 KB) [file 213_2021_6047_MOESM1_ESM.docx]

**Supplementary Materials**

**Prevalence and self-reported reasons of cannabis use for medical purposes in USA and Canada**

Janni Leung^1,2^, Gary Chan^1^, Daniel Stjepanović^1^, Jack Yiu Chak Chung^1,2^, Wayne Hall^1^, David Hammond^3^

1. National Centre for Youth Substance Use Research, The University of Queensland, Australia
2. School of Psychology, The University of Queensland, Australia
3. School of Public Health and Health Systems, University of Waterloo, Canada

**Table of Contents**

[S1. Cannabis policy conditions defined by recreational and medical cannabis laws in 2018 2](#_Toc89869134)

[S2. Data tables for prevalence of self-reported ever cannabis use for medical purposes by cannabis policy conditions of jurisdiction by gender and age groups 4](#_Toc89869135)

[Table S2.1. Weighted self-reported prevalence of cannabis use for medical reasons by cannabis policy condition by gender and age 4](#_Toc89869136)

[Table S2.2. Unweighted prevalence of self-reported ever cannabis use for medical purposes by cannabis policy condition by gender and age 6](#_Toc89869137)

[S3. Prevalence of reasons for which cannabis use for medical purposes was ever used to manage 7](#_Toc89869138)

[Table S3. Unweighted and weighted prevalence of reasons for which cannabis was ever used for medical purposes, among people who self-reported ever cannabis use for medical purposes 7](#_Toc89869139)

[S4. Spearman’s correlation between self-reported medical reasons for which cannabis was ever use 8](#_Toc89869140)

[S5. Multiple logistic regression on self-reported ever cannabis use for medical purposes for each of the reasons of use. 9](#_Toc89869141)

[S6. Control variables in the multiple logistic regressions on self-reported ever cannabis use for medical reasons 13](#_Toc89869142)

[S7 Proportion of International Cannabis Policy Study (ICPS) 2018 respondents by province or state of residencea (n=27,169) 16](#_Toc89869143)

# S1. Cannabis policy conditions defined by recreational and medical cannabis laws in 2018

| **Country** | **Jurisdiction** | **Recreational use policy condition** | **Medical market policy condition** | **Cannabis policy condition operationalized†** |
| --- | --- | --- | --- | --- |
| Canada | All jurisdictions | Canada - illegal | Legal medical market | Canada - medical only |
| United States | Alabama | US illegal state | No legal medical market | US illegal |
| United States | Alaska | US legal state | Legal medical market | US legal - recreational |
| United States | Arizona | US illegal state | Legal medical market | US legal - medical only |
| United States | Arkansas | US illegal state | Legal medical market | US legal - medical only |
| United States | California | US legal state | Legal medical market | US legal - recreational |
| United States | Colorado | US legal state | Legal medical market | US legal - recreational |
| United States | Connecticut | US illegal state | Legal medical market | US legal - medical only |
| United States | Delaware | US illegal state | Legal medical market | US legal - medical only |
| United States | Florida | US illegal state | Legal medical market | US legal - medical only |
| United States | Georgia | US illegal state | No legal medical market | US illegal |
| United States | Hawaii | US illegal state | Legal medical market | US legal - medical only |
| United States | Idaho | US illegal state | No legal medical market | US illegal |
| United States | Illinois | US illegal state | Legal medical market | US legal - medical only |
| United States | Indiana | US illegal state | No legal medical market | US illegal |
| United States | Iowa | US illegal state | No legal medical market | US illegal |
| United States | Kansas | US illegal state | No legal medical market | US illegal |
| United States | Kentucky | US illegal state | No legal medical market | US illegal |
| United States | Louisiana | US illegal state | Legal medical market | US legal - medical only |
| United States | Maine | US legal state | Legal medical market | US legal - recreational |
| United States | Maryland | US illegal state | Legal medical market | US legal - medical only |
| United States | Massachusetts | US legal state | Legal medical market | US legal - recreational |
| United States | Michigan | US illegal state | Legal medical market | US legal - medical only |
| United States | Minnesota | US illegal state | Legal medical market | US legal - medical only |
| United States | Mississippi | US illegal state | No legal medical market | US illegal |
| United States | Missouri | US illegal state | Legal medical market | US legal - medical only |
| United States | Montana | US illegal state | Legal medical market | US legal - medical only |
| United States | Nebraska | US illegal state | No legal medical market | US illegal |
| United States | Nevada | US legal state | Legal medical market | US legal - recreational |
| United States | New Hampshire | US illegal state | Legal medical market | US legal - medical only |
| United States | New Jersey | US illegal state | Legal medical market | US legal - medical only |
| United States | New Mexico | US illegal state | Legal medical market | US legal - medical only |
| United States | New York | US illegal state | Legal medical market | US legal - medical only |
| United States | North Carolina | US illegal state | No legal medical market | US illegal |
| United States | North Dakota | US illegal state | Legal medical market | US legal - medical only |
| United States | Ohio | US illegal state | Legal medical market | US legal - medical only |
| United States | Oklahoma | US illegal state | Legal medical market | US legal - medical only |
| United States | Oregon | US legal state | Legal medical market | US legal - recreational |
| United States | Pennsylvania | US illegal state | Legal medical market | US legal - medical only |
| United States | Rhode Island | US illegal state | Legal medical market | US legal - medical only |
| United States | South Carolina | US illegal state | No legal medical market | US illegal |
| United States | South Dakota | US illegal state | No legal medical market | US illegal |
| United States | Tennessee | US illegal state | No legal medical market | US illegal |
| United States | Texas | US illegal state | No legal medical market | US illegal |
| United States | Utah | US illegal state | Legal medical market | US legal - medical only |
| United States | Vermont | US legal state | Legal medical market | US legal - recreational |
| United States | Virginia | US illegal state | No legal medical market | US illegal |
| United States | Washington State | US legal state | Legal medical market | US legal - recreational |
| United States | West Virginia | US illegal state | Legal medical market | US legal - medical only |
| United States | Wisconsin | US illegal state | No legal medical market | US illegal |
| United States | Wyoming | US illegal state | No legal medical market | US illegal |
| *Note.* Categories were defined at the time of survey in 2018;  †Variable used in analysis. In the USA, all the states that had legalized recreational cannabis had also legalized medical cannabis. Therefore, the three US policy conditions operationalized were:  (1) “US legal – recreational”: legal for both recreational and medical,  (2) “US legal – medical only”: legal medical market, but illegal for recreational use,  (3) “US – illegal”: illegal for recreational use and no legal medical market | | | | |

# S2. Data tables for prevalence of self-reported ever cannabis use for medical purposes by cannabis policy conditions of jurisdiction by gender and age groups

## Table S2.1. Weighted self-reported prevalence of cannabis use for medical reasons by cannabis policy condition by gender and age

|  |  |  | **Sample size** | **Weighted prevalence of self-reported medical cannabis use by Cannabis policy condition of jurisdiction** | | | | | | | | | | | |
| --- | --- | --- | --- | --- | --- | --- | --- | --- | --- | --- | --- | --- | --- | --- | --- |
|  |  |  |  | **Canada - medical only** | | | **US legal - recreational** | | | **US legal - medical only** | | | **US illegal** | | |
|  |  |  |  | **%** | **lower** | **upper** | **%** | **lower** | **upper** | **%** | **lower** | **upper** | **%** | **lower** | **upper** |
| **By gender identity†** | | |  |  |  |  |  |  |  |  |  |  |  |  |  |
|  | Female | | 16508 | 25% | 24% | 25% | 34% | 33% | 35% | 24% | 23% | 24% | 21% | 20% | 22% |
|  | Male | | 10404 | 24% | 24% | 25% | 33% | 32% | 34% | 26% | 26% | 27% | 25% | 24% | 26% |
| **By age** | | |  |  |  |  |  |  |  |  |  |  |  |  |  |
|  | 16-25 | | 4296 | 22% | 21% | 23% | 26% | 25% | 28% | 19% | 18% | 20% | 17% | 15% | 18% |
|  | 26-35 | | 4011 | 35% | 34% | 37% | 47% | 45% | 48% | 34% | 33% | 36% | 32% | 30% | 33% |
|  | 36-45 | | 4290 | 25% | 24% | 26% | 37% | 35% | 38% | 28% | 26% | 29% | 26% | 24% | 27% |
|  | 46-55 | | 5638 | 21% | 20% | 23% | 30% | 29% | 31% | 26% | 25% | 27% | 23% | 22% | 24% |
|  | 56-65 | | 8934 | 19% | 18% | 20% | 28% | 27% | 29% | 19% | 18% | 19% | 17% | 16% | 17% |
| **By gender and age** | | |  |  |  |  |  |  |  |  |  |  |  |  |  |
|  | **Female** | |  |  |  |  |  |  |  |  |  |  |  |  |  |
|  |  | 16-25 | 2453 | 23% | 21% | 25% | 27% | 26% | 29% | 21% | 19% | 23% | 17% | 15% | 18% |
|  |  | 26-35 | 2597 | 33% | 32% | 35% | 45% | 43% | 47% | 30% | 29% | 32% | 29% | 28% | 31% |
|  |  | 36-45 | 2713 | 26% | 24% | 27% | 36% | 34% | 38% | 26% | 24% | 28% | 21% | 19% | 23% |
|  |  | 46-55 | 3652 | 24% | 23% | 25% | 34% | 32% | 35% | 25% | 23% | 26% | 23% | 21% | 24% |
|  |  | 56-65 | 5093 | 17% | 16% | 18% | 27% | 26% | 28% | 17% | 16% | 18% | 14% | 13% | 15% |
|  | **Male** | |  |  |  |  |  |  |  |  |  |  |  |  |  |
|  |  | 16-25 | 1744 | 20% | 18% | 22% | 24% | 22% | 26% | 17% | 16% | 19% | 16% | 14% | 18% |
|  |  | 26-35 | 1354 | 37% | 35% | 40% | 47% | 44% | 50% | 37% | 34% | 40% | 34% | 31% | 37% |
|  |  | 36-45 | 1537 | 25% | 23% | 27% | 36% | 34% | 39% | 29% | 27% | 32% | 31% | 29% | 33% |
|  |  | 46-55 | 1954 | 19% | 17% | 20% | 26% | 24% | 28% | 27% | 25% | 29% | 24% | 22% | 26% |
|  |  | 56-65 | 3815 | 21% | 20% | 22% | 30% | 28% | 31% | 20% | 19% | 21% | 19% | 18% | 21% |
| †Other gender identity not reported due to low frequencies; Cannabis policy conditions were defined at the time of survey in 2018; In the USA, all the states that had legalized recreational cannabis had also legalized medical cannabis. Therefore, the three US policy conditions operationalized were: (1) “US legal – recreational”: legal for both recreational and medical, (2) “US legal – medical only”: legal medical market, but illegal for recreational use, (3) “US – illegal”: illegal for recreational use and no legal medical market | | | | | | | | | | | | | | | |

## Table S2.2. Unweighted prevalence of self-reported ever cannabis use for medical purposes by cannabis policy condition by gender and age

|  |  |  | **Sample size** | **Unweighted prevalence of self-reported cannabis use for medical purposes**  **by Cannabis policy condition of jurisdiction** | | | | | | | | | | | |
| --- | --- | --- | --- | --- | --- | --- | --- | --- | --- | --- | --- | --- | --- | --- | --- |
|  |  |  |  | **Canada - medical only** | | | **US legal - recreational** | | | **US legal - medical only** | | | **US illegal** | | |
|  |  |  |  | **%** | lower | upper | **%** | lower | upper | **%** | lower | upper | **%** | lower | upper |
| **By gender identity†** | | |  |  |  |  |  |  |  |  |  |  |  |  |  |
|  | Female | | 16508 | 23% | 22% | 23% | 32% | 32% | 33% | 21% | 21% | 22% | 19% | 18% | 19% |
|  | Male | | 10404 | 22% | 21% | 22% | 30% | 29% | 31% | 23% | 22% | 24% | 21% | 20% | 22% |
| **By age** | | |  |  |  |  |  |  |  |  |  |  |  |  |  |
|  | 16-25 | | 4296 | 26% | 25% | 27% | 33% | 31% | 34% | 18% | 17% | 19% | 16% | 15% | 17% |
|  | 26-35 | | 4011 | 34% | 32% | 35% | 43% | 41% | 44% | 32% | 30% | 33% | 27% | 26% | 29% |
|  | 36-45 | | 4290 | 24% | 22% | 25% | 34% | 33% | 36% | 25% | 24% | 27% | 24% | 23% | 25% |
|  | 46-55 | | 5638 | 22% | 20% | 23% | 29% | 28% | 30% | 23% | 22% | 24% | 21% | 20% | 22% |
|  | 56-65 | | 8934 | 17% | 16% | 17% | 26% | 25% | 27% | 18% | 17% | 18% | 16% | 15% | 16% |
| **By gender and age** | | |  |  |  |  |  |  |  |  |  |  |  |  |  |
|  | **Female** | |  |  |  |  |  |  |  |  |  |  |  |  |  |
|  |  | 16-25 | 2453 | 26% | 24% | 27% | 35% | 33% | 37% | 20% | 18% | 21% | 17% | 15% | 18% |
|  |  | 26-35 | 2597 | 32% | 31% | 34% | 41% | 39% | 43% | 29% | 27% | 31% | 26% | 25% | 28% |
|  |  | 36-45 | 2713 | 25% | 24% | 27% | 35% | 33% | 37% | 23% | 21% | 24% | 20% | 19% | 22% |
|  |  | 46-55 | 3652 | 24% | 23% | 26% | 30% | 29% | 32% | 22% | 21% | 23% | 21% | 20% | 22% |
|  |  | 56-65 | 5093 | 15% | 14% | 16% | 27% | 26% | 28% | 17% | 16% | 18% | 14% | 13% | 14% |
|  | **Male** | |  |  |  |  |  |  |  |  |  |  |  |  |  |
|  |  | 16-25 | 1744 | 25% | 23% | 27% | 26% | 24% | 28% | 16% | 15% | 18% | 15% | 13% | 16% |
|  |  | 26-35 | 1354 | 36% | 33% | 38% | 46% | 43% | 49% | 37% | 34% | 39% | 29% | 27% | 31% |
|  |  | 36-45 | 1537 | 22% | 19% | 24% | 32% | 30% | 35% | 29% | 27% | 31% | 32% | 30% | 34% |
|  |  | 46-55 | 1954 | 18% | 16% | 19% | 26% | 24% | 28% | 26% | 24% | 28% | 23% | 21% | 24% |
|  |  | 56-65 | 3815 | 18% | 17% | 19% | 25% | 24% | 27% | 18% | 17% | 19% | 18% | 17% | 19% |
| †Other gender identity not reported due to low frequencies; Cannabis policy conditions were defined at the time of survey in 2018; In the USA, all the states that had legalized recreational cannabis had also legalized medical cannabis. Therefore, the three US policy conditions operationalized were: (1) “US legal – recreational”: legal for both recreational and medical, (2) “US legal – medical only”: legal medical market, but illegal for recreational use, (3) “US – illegal”: illegal for recreational use and no legal medical market | | | | | | | | | | | | | | | |

# S3. Prevalence of reasons for which cannabis use for medical purposes was ever used to manage

## Table S3. Unweighted and weighted prevalence of reasons for which cannabis was ever used for medical purposes, among people who self-reported ever cannabis use for medical purposes

|  |  | Unweighted | | | | Weighted | | |
| --- | --- | --- | --- | --- | --- | --- | --- | --- |
|  |  | n | % | lower | upper | % | lower | upper |
| Used for medical conditions | |  |  |  |  |  |  |  |
|  | Headaches/migraines | 2243 | 34% | 33% | 35% | 35% | 34% | 36% |
|  | Pain | 3761 | 57% | 55% | 58% | 53% | 52% | 55% |
|  | Nausea/vomiting | 1399 | 21% | 20% | 22% | 21% | 20% | 22% |
|  | Lack of appetite | 1264 | 19% | 18% | 20% | 22% | 21% | 23% |
|  | Seizures | 230 | 3% | 3% | 4% | 5% | 4% | 5% |
|  | Muscle spasms | 876 | 13% | 12% | 14% | 14% | 13% | 15% |
|  | Cancer | 184 | 3% | 2% | 3% | 3% | 3% | 3% |
|  | Problems sleeping | 3179 | 48% | 47% | 49% | 46% | 45% | 47% |
| Used for mental health | |  |  |  |  |  |  |  |
|  | Anxiety | 3371 | 51% | 50% | 52% | 52% | 51% | 53% |
|  | Depression | 2439 | 37% | 36% | 38% | 40% | 38% | 41% |
|  | Post-traumatic stress | 1038 | 16% | 15% | 17% | 17% | 16% | 17% |
|  | Bipolar disorder | 522 | 8% | 7% | 9% | 10% | 9% | 10% |
|  | Psychosis | 191 | 3% | 2% | 3% | 4% | 4% | 5% |
|  | Schizophrenia | 123 | 2% | 2% | 2% | 3% | 3% | 3% |
|  | Drug or alcohol use | 608 | 9% | 8% | 10% | 11% | 10% | 12% |

# S4. Spearman’s correlation between self-reported medical reasons for which cannabis was ever use

|  |  | **1** | **2** | **3** | **4** | **5** | **6** | **7** | **8** | **9** | **10** | **11** | **12** | **13** | **14** |
| --- | --- | --- | --- | --- | --- | --- | --- | --- | --- | --- | --- | --- | --- | --- | --- |
| **1** | Headaches/migraines | -- |  |  |  |  |  |  |  |  |  |  |  |  |  |
| **2** | Pain | .43 | -- |  |  |  |  |  |  |  |  |  |  |  |  |
| **3** | Nausea/vomiting | .37 | .36 | -- |  |  |  |  |  |  |  |  |  |  |  |
| **4** | Lack of appetite | .36 | .38 | .46 | -- |  |  |  |  |  |  |  |  |  |  |
| **5** | Seizures | .16 | .13 | .19 | .14 | -- |  |  |  |  |  |  |  |  |  |
| **6** | Muscle spasms | .28 | .35 | .27 | .27 | .17 | -- |  |  |  |  |  |  |  |  |
| **7** | Cancer/tumours | .10 | .10 | .14 | .14 | .24 | .12 | -- |  |  |  |  |  |  |  |
| **8** | Problems sleeping | .39 | .48 | .36 | .42 | .08 | .27 | .05 | -- |  |  |  |  |  |  |
| **9** | Anxiety | .47 | .47 | .37 | .38 | .14 | .25 | .09 | .48 | -- |  |  |  |  |  |
| **10** | Depression | .43 | .46 | .38 | .42 | .15 | .28 | .11 | .45 | .61 | -- |  |  |  |  |
| **11** | PTSD/trauma | .27 | .33 | .34 | .34 | .16 | .27 | .11 | .31 | .38 | .38 | -- |  |  |  |
| **12** | Bipolar/mania | .22 | .22 | .21 | .23 | .21 | .22 | .16 | .19 | .28 | .32 | .21 | -- |  |  |
| **13** | Psychosis | .14 | .14 | .16 | .20 | .21 | .20 | .14 | .15 | .17 | .19 | .25 | .24 | -- |  |
| **14** | Schizophrenia | .12 | .07 | .10 | .12 | .17 | .17 | .19 | .07 | .09 | .12 | .18 | .24 | .32 | -- |
| **15** | Drug/alcohol use | .19 | .20 | .19 | .24 | .09 | .13 | .08 | .25 | .21 | .26 | .19 | .17 | .18 | .09 |

# S5. Multiple logistic regression on self-reported ever cannabis use for medical purposes for each of the reasons of use.

| **Separate models fitted for each of the reason outcome†** | | | **Adjusted odds ratios** | | | |
| --- | --- | --- | --- | --- | --- | --- |
|  |  |  | **OR** | **95%CI** | |  |
|  |  |  |  | **lower** | **upper** | **p** |
| **Use for Headaches/migraines** | | |  |  |  |  |
|  | Male vs Female |  | 0.84 | 0.77 | 0.92 | <0.001 |
|  | Age (ref: 16-25) | 26-35 | 1.81 | 1.57 | 2.09 | <0.001 |
|  |  | 36-45 | 0.98 | 0.84 | 1.14 | 0.789 |
|  |  | 46-55 | 0.54 | 0.46 | 0.64 | <0.001 |
|  |  | 56-65 | 0.30 | 0.25 | 0.36 | <0.001 |
|  | Cannabis policy condition (ref: US illegal) | Canada - medical only | 1.01 | 0.87 | 1.16 | 0.921 |
|  |  | US legal - recreational | 1.62 | 1.41 | 1.87 | <0.001 |
|  |  | US legal - medical only | 1.20 | 1.03 | 1.40 | 0.021 |
| **Use for Pain, including Other: Arthritis, Neuropathy or Menstrual cramps/PMS^‡^** | | |  |  |  |  |
|  | Male vs Female |  | 0.95 | 0.88 | 1.02 | 0.152 |
|  | Age (ref: 16-25) | 26-35 | 2.57 | 2.22 | 2.98 | <0.001 |
|  |  | 36-45 | 1.93 | 1.67 | 2.24 | <0.001 |
|  |  | 46-55 | 1.68 | 1.45 | 1.95 | <0.001 |
|  |  | 56-65 | 1.46 | 1.26 | 1.69 | <0.001 |
|  | Cannabis policy condition (ref: US illegal) | Canada - medical only | 1.31 | 1.16 | 1.48 | <0.001 |
|  |  | US legal - recreational | 2.04 | 1.81 | 2.31 | <0.001 |
|  |  | US legal - medical only | 1.35 | 1.18 | 1.54 | <0.001 |
| **Use for Nausea/vomiting, or Other: Chemotherapy** | | |  |  |  |  |
|  | Male vs Female |  | 0.73 | 0.65 | 0.81 | <0.001 |
|  | Age (ref: 16-25) | 26-35 | 1.91 | 1.59 | 2.30 | <0.001 |
|  |  | 36-45 | 1.32 | 1.08 | 1.61 | 0.007 |
|  |  | 46-55 | 0.73 | 0.59 | 0.90 | 0.003 |
|  |  | 56-65 | 0.49 | 0.39 | 0.62 | <0.001 |
|  | Cannabis policy condition (ref: US illegal) | Canada - medical only | 0.77 | 0.65 | 0.93 | 0.005 |
|  |  | US legal - recreational | 1.46 | 1.23 | 1.74 | <0.001 |
|  |  | US legal - medical only | 1.08 | 0.89 | 1.30 | 0.445 |
| **Use for Lack of appetite** | | |  |  |  |  |
|  | Male vs Female |  | 0.97 | 0.87 | 1.08 | 0.582 |
|  | Age (ref: 16-25) | 26-35 | 1.79 | 1.50 | 2.14 | <0.001 |
|  |  | 36-45 | 1.13 | 0.93 | 1.36 | 0.223 |
|  |  | 46-55 | 0.69 | 0.56 | 0.84 | <0.001 |
|  |  | 56-65 | 0.51 | 0.41 | 0.63 | <0.001 |
|  | Cannabis policy condition (ref: US illegal) | Canada - medical only | 1.21 | 1.01 | 1.45 | 0.041 |
|  |  | US legal - recreational | 1.85 | 1.54 | 2.23 | <0.001 |
|  |  | US legal - medical only | 1.30 | 1.07 | 1.58 | 0.010 |
| **Use for Seizures** | | |  |  |  |  |
|  | Male vs Female |  | 1.62 | 1.29 | 2.04 | <0.001 |
|  | Age (ref: 16-25) | 26-35 | 1.90 | 1.27 | 2.83 | 0.002 |
|  |  | 36-45 | 1.39 | 0.91 | 2.11 | 0.126 |
|  |  | 46-55 | 0.61 | 0.39 | 0.97 | 0.036 |
|  |  | 56-65 | 0.23 | 0.12 | 0.44 | <0.001 |
|  | Cannabis policy condition (ref: US illegal) | Canada - medical only | 0.71 | 0.48 | 1.03 | 0.071 |
|  |  | US legal - recreational | 1.84 | 1.27 | 2.67 | 0.001 |
|  |  | US legal - medical only | 0.65 | 0.42 | 1.02 | 0.062 |
| **Use for Muscle spasms** | | |  |  |  |  |
|  | Male vs Female |  | 1.09 | 0.96 | 1.24 | 0.195 |
|  | Age (ref: 16-25) | 26-35 | 4.04 | 2.98 | 5.47 | <0.001 |
|  |  | 36-45 | 3.42 | 2.50 | 4.67 | <0.001 |
|  |  | 46-55 | 2.61 | 1.91 | 3.56 | <0.001 |
|  |  | 56-65 | 1.84 | 1.33 | 2.56 | <0.001 |
|  | Cannabis policy condition (ref: US illegal) | Canada - medical only | 0.86 | 0.69 | 1.07 | 0.172 |
|  |  | US legal - recreational | 1.54 | 1.24 | 1.91 | <0.001 |
|  |  | US legal - medical only | 1.23 | 0.98 | 1.54 | 0.076 |
| **Use to treat cancer, including Other: To treat tumours** | | |  |  |  |  |
|  | Male vs Female |  | 1.67 | 1.26 | 2.21 | <0.001 |
|  | Age (ref: 16-25) | 26-35 | 4.08 | 2.05 | 8.13 | <0.001 |
|  |  | 36-45 | 2.92 | 1.41 | 6.02 | 0.004 |
|  |  | 46-55 | 2.22 | 1.06 | 4.66 | 0.035 |
|  |  | 56-65 | 1.99 | 0.94 | 4.23 | 0.074 |
|  | Cannabis policy condition (ref: US illegal) | Canada - medical only | 0.73 | 0.47 | 1.12 | 0.148 |
|  |  | US legal - recreational | 1.10 | 0.71 | 1.70 | 0.667 |
|  |  | US legal - medical only | 1.07 | 0.68 | 1.68 | 0.767 |
| **Use for Problems sleeping** | | |  |  |  |  |
|  | Male vs Female |  | 0.90 | 0.84 | 0.97 | 0.008 |
|  | Age (ref: 16-25) | 26-35 | 1.79 | 1.55 | 2.07 | <0.001 |
|  |  | 36-45 | 1.31 | 1.13 | 1.52 | <0.001 |
|  |  | 46-55 | 1.09 | 0.95 | 1.26 | 0.217 |
|  |  | 56-65 | 0.87 | 0.75 | 1.01 | 0.065 |
|  | Cannabis policy condition (ref: US illegal) | Canada - medical only | 1.44 | 1.26 | 1.65 | <0.001 |
|  |  | US legal - recreational | 1.99 | 1.72 | 2.30 | <0.001 |
|  |  | US legal - medical only | 1.31 | 1.13 | 1.52 | <0.001 |
| **Use for Anxiety (including phobia, obsessive-compulsive disorder or a panic disorder)** | | |  |  |  |  |
|  | Male vs Female |  | 0.88 | 0.82 | 0.95 | <0.001 |
|  | Age (ref: 16-25) | 26-35 | 1.53 | 1.36 | 1.73 | <0.001 |
|  |  | 36-45 | 0.94 | 0.83 | 1.07 | 0.387 |
|  |  | 46-55 | 0.63 | 0.55 | 0.72 | <0.001 |
|  |  | 56-65 | 0.34 | 0.30 | 0.40 | <0.001 |
|  | Cannabis policy condition (ref: US illegal) | Canada - medical only | 0.91 | 0.81 | 1.02 | 0.098 |
|  |  | US legal - recreational | 1.38 | 1.23 | 1.55 | <0.001 |
|  |  | US legal - medical only | 1.06 | 0.94 | 1.20 | 0.330 |
| **Use for Depression (including dysthymia)** | | |  |  |  |  |
|  | Male vs Female |  | 1.01 | 0.93 | 1.09 | 0.876 |
|  | Age (ref: 16-25) | 26-35 | 1.71 | 1.50 | 1.96 | <0.001 |
|  |  | 36-45 | 1.05 | 0.91 | 1.22 | 0.474 |
|  |  | 46-55 | 0.61 | 0.52 | 0.71 | <0.001 |
|  |  | 56-65 | 0.41 | 0.35 | 0.48 | <0.001 |
|  | Cannabis policy condition (ref: US illegal) | Canada - medical only | 1.10 | 0.97 | 1.26 | 0.152 |
|  |  | US legal - recreational | 1.44 | 1.25 | 1.65 | <0.001 |
|  |  | US legal - medical only | 1.19 | 1.03 | 1.37 | 0.018 |
| **Use for Post-traumatic stress disorder (PTSD) or traumatic event (e.g., abuse or loss)** | | |  |  |  |  |
|  | Male vs Female |  | 0.81 | 0.72 | 0.92 | <0.001 |
|  | Age (ref: 16-25) | 26-35 | 2.09 | 1.67 | 2.60 | <0.001 |
|  |  | 36-45 | 1.71 | 1.36 | 2.15 | <0.001 |
|  |  | 46-55 | 0.88 | 0.69 | 1.13 | 0.317 |
|  |  | 56-65 | 0.62 | 0.48 | 0.80 | <0.001 |
|  | Cannabis policy condition (ref: US illegal) | Canada - medical only | 1.09 | 0.88 | 1.34 | 0.447 |
|  |  | US legal - recreational | 1.95 | 1.59 | 2.41 | <0.001 |
|  |  | US legal - medical only | 1.32 | 1.05 | 1.65 | 0.016 |
| **Use for Bipolar disorder or mania, including Other: Borderline Personality disorder** | | |  |  |  |  |
|  | Male vs Female |  | 1.27 | 1.08 | 1.49 | 0.003 |
|  | Age (ref: 16-25) | 26-35 | 1.78 | 1.36 | 2.34 | <0.001 |
|  |  | 36-45 | 1.26 | 0.95 | 1.67 | 0.111 |
|  |  | 46-55 | 0.78 | 0.58 | 1.05 | 0.101 |
|  |  | 56-65 | 0.26 | 0.17 | 0.39 | <0.001 |
|  | Cannabis policy condition (ref: US illegal) | Canada - medical only | 0.80 | 0.61 | 1.05 | 0.102 |
|  |  | US legal - recreational | 1.50 | 1.16 | 1.95 | 0.002 |
|  |  | US legal - medical only | 1.58 | 1.21 | 2.07 | <0.001 |
| **Use for Psychosis (e.g., paranoia, disorganized thinking, hearing voices that others can't** | | |  |  |  |  |
|  | Male vs Female |  | 2.72 | 2.09 | 3.54 | <0.001 |
|  | Age (ref: 16-25) | 26-35 | 2.30 | 1.48 | 3.57 | <0.001 |
|  |  | 36-45 | 1.83 | 1.16 | 2.89 | 0.009 |
|  |  | 46-55 | 0.72 | 0.42 | 1.23 | 0.231 |
|  |  | 56-65 | 0.29 | 0.15 | 0.55 | <0.001 |
|  | Cannabis policy condition (ref: US illegal) | Canada - medical only | 0.90 | 0.59 | 1.38 | 0.624 |
|  |  | US legal - recreational | 1.98 | 1.31 | 3.01 | 0.001 |
|  |  | US legal - medical only | 1.07 | 0.69 | 1.67 | 0.767 |
| **Use for Schizophrenia** | | |  |  |  |  |
|  | Male vs Female |  | 4.02 | 2.86 | 5.67 | <0.001 |
|  | Age (ref: 16-25) | 26-35 | 3.42 | 2.03 | 5.76 | <0.001 |
|  |  | 36-45 | 2.32 | 1.33 | 4.03 | 0.003 |
|  |  | 46-55 | 0.41 | 0.18 | 0.92 | 0.032 |
|  |  | 56-65 | 0.33 | 0.15 | 0.73 | 0.006 |
|  | Cannabis policy condition (ref: US illegal) | Canada - medical only | 0.80 | 0.51 | 1.25 | 0.332 |
|  |  | US legal - recreational | 1.42 | 0.93 | 2.18 | 0.108 |
|  |  | US legal - medical only | 0.55 | 0.32 | 0.94 | 0.027 |
| **Use for Drug or alcohol use** | | |  |  |  |  |
|  | Male vs Female |  | 1.58 | 1.36 | 1.83 | <0.001 |
|  | Age (ref: 16-25) | 26-35 | 1.96 | 1.51 | 2.54 | <0.001 |
|  |  | 36-45 | 1.36 | 1.03 | 1.79 | 0.030 |
|  |  | 46-55 | 1.03 | 0.78 | 1.36 | 0.844 |
|  |  | 56-65 | 0.54 | 0.39 | 0.75 | <0.001 |
|  | Cannabis policy condition (ref: US illegal) | Canada - medical only | 1.20 | 0.93 | 1.54 | 0.158 |
|  |  | US legal - recreational | 1.23 | 0.96 | 1.60 | 0.106 |
|  |  | US legal - medical only | 1.41 | 1.09 | 1.83 | 0.009 |
| †Use for each of the reasons were analysed in separate models; all models adjusted for gender, age, cannabis policy condition, ethnicity, and education (see S6 for results for control variables).  **^‡^**Menstrual cramps/PMS was included in the pain group, however, it was only reported by N=15 participants and therefore unlikely to explain the gender result. | | | | | | |

# S6. Control variables in the multiple logistic regressions on self-reported ever cannabis use for medical reasons

| **Separate models fitted for any reasons, then each of the reason outcome†** | | | **Adjusted odds ratios** | | | |  |
| --- | --- | --- | --- | --- | --- | --- | --- |
|  |  |  | **OR** | **95%CI** | |  |  |
|  |  |  |  | **lower** | **upper** | **p** |  |
| **Use for Any reasons** | | |  |  |  |  |  |
|  | Ethnicity (other vs white) | | 0.80 | 0.75 | 0.86 | <0.001 |  |
|  | Education (ref: less than high school) | High school diploma or equivalent | 1.44 | 1.28 | 1.62 | <0.001 |  |
|  |  | Some college | 1.57 | 1.40 | 1.76 | <0.001 |  |
|  |  | Bachelor or higher | 0.82 | 0.73 | 0.93 | 0.002 |  |
| **Use for Headaches/migraines** | | |  |  |  |  |  |
|  | Ethnicity (other vs white) | | 0.80 | 0.72 | 0.89 | <0.001 |  |
|  | Education (ref: less than high school) | High school diploma or equivalent | 2.11 | 1.77 | 2.51 | <0.001 |  |
|  |  | Some college | 2.06 | 1.73 | 2.45 | <0.001 |  |
|  |  | Bachelor or higher | 1.26 | 1.04 | 1.51 | 0.016 |  |
| **Use for Pain, including Other: Arthritis, Neuropathy or Menstrual cramps/PMS** | | |  |  |  |  |  |
|  | Ethnicity (other vs white) | | 0.85 | 0.78 | 0.93 | <0.001 |  |
|  | Education (ref: less than high school) | High school diploma or equivalent | 1.42 | 1.21 | 1.67 | <0.001 |  |
|  |  | Some college | 1.50 | 1.28 | 1.77 | <0.001 |  |
|  |  | Bachelor or higher | 0.64 | 0.54 | 0.76 | <0.001 |  |
| **Use for Nausea/vomiting, or Other: Chemotherapy** | | |  |  |  |  |  |
|  | Ethnicity (other vs white) | | 0.88 | 0.77 | 1.00 | 0.054 |  |
|  | Education (ref: less than high school) | High school diploma or equivalent | 2.23 | 1.76 | 2.84 | <0.001 |  |
|  |  | Some college | 2.11 | 1.67 | 2.67 | <0.001 |  |
|  |  | Bachelor or higher | 1.12 | 0.87 | 1.43 | 0.384 |  |
| **Use for Lack of appetite** | | |  |  |  |  |  |
|  | Ethnicity (other vs white) | | 1.13 | 1.00 | 1.27 | 0.055 |  |
|  | Education (ref: less than high school) | High school diploma or equivalent | 1.72 | 1.39 | 2.14 | <0.001 |  |
|  |  | Some college | 1.76 | 1.43 | 2.16 | <0.001 |  |
|  |  | Bachelor or higher | 0.74 | 0.59 | 0.93 | 0.011 |  |
| **Use for Seizures** | | |  |  |  |  |  |
|  | Ethnicity (other vs white) | | 0.98 | 0.75 | 1.28 | 0.865 |  |
|  | Education (ref: less than high school) | High school diploma or equivalent | 3.21 | 1.66 | 6.20 | <0.001 |  |
|  |  | Some college | 2.89 | 1.54 | 5.42 | 0.001 |  |
|  |  | Bachelor or higher | 2.91 | 1.51 | 5.64 | 0.002 |  |
| **Use for Muscle spasms** | | |  |  |  |  |  |
|  | Ethnicity (other vs white) | | 1.13 | 0.97 | 1.32 | 0.126 |  |
|  | Education (ref: less than high school) | High school diploma or equivalent | 1.91 | 1.36 | 2.70 | <0.001 |  |
|  |  | Some college | 1.73 | 1.24 | 2.40 | 0.001 |  |
|  |  | Bachelor or higher | 1.06 | 0.75 | 1.49 | 0.739 |  |
| **Use for To treat cancer, including Other: To treat tumours** | | |  |  |  |  |  |
|  | Ethnicity (other vs white) | | 0.81 | 0.57 | 1.15 | 0.236 |  |
|  | Education (ref: less than high school) | High school diploma or equivalent | 2.41 | 1.05 | 5.54 | 0.038 |  |
|  |  | Some college | 2.60 | 1.14 | 5.95 | 0.023 |  |
|  |  | Bachelor or higher | 1.47 | 0.64 | 3.42 | 0.365 |  |
| **Use for Problems sleeping** | | |  |  |  |  |  |
|  | Ethnicity (other vs white) | | 0.81 | 0.73 | 0.89 | <0.001 |  |
|  | Education (ref: less than high school) | High school diploma or equivalent | 1.65 | 1.40 | 1.93 | <0.001 |  |
|  |  | Some college | 1.61 | 1.37 | 1.89 | <0.001 |  |
|  |  | Bachelor or higher | 0.73 | 0.61 | 0.87 | <0.001 |  |
| **Use for Anxiety (including phobia, obsessive-compulsive disorder or a panic disorder)** | | |  |  |  |  |  |
|  | Ethnicity (other vs white) | | 0.68 | 0.62 | 0.75 | <0.001 |  |
|  | Education (ref: less than high school) | High school diploma or equivalent | 1.49 | 1.28 | 1.72 | <0.001 |  |
|  |  | Some college | 1.65 | 1.44 | 1.90 | <0.001 |  |
|  |  | Bachelor or higher | 0.92 | 0.79 | 1.07 | 0.267 |  |
| **Use for Depression (including dysthymia)** | | |  |  |  |  |  |
|  | Ethnicity (other vs white) | | 0.87 | 0.79 | 0.96 | 0.007 |  |
|  | Education (ref: less than high school) | High school diploma or equivalent | 2.04 | 1.73 | 2.41 | <0.001 |  |
|  |  | Some college | 1.88 | 1.60 | 2.22 | <0.001 |  |
|  |  | Bachelor or higher | 0.96 | 0.81 | 1.15 | 0.665 |  |
| **Use for Post-traumatic stress disorder (PTSD) or traumatic event (e.g., abuse or loss)** | | |  |  |  |  |  |
|  | Ethnicity (other vs white) | | 0.94 | 0.81 | 1.09 | 0.419 |  |
|  | Education (ref: less than high school) | High school diploma or equivalent | 1.56 | 1.19 | 2.04 | 0.001 |  |
|  |  | Some college | 1.97 | 1.53 | 2.56 | <0.001 |  |
|  |  | Bachelor or higher | 1.14 | 0.86 | 1.50 | 0.363 |  |
| **Use for Bipolar disorder or mania, including Other: Borderline Personality disorder** | | |  |  |  |  |  |
|  | Ethnicity (other vs white) | | 0.99 | 0.82 | 1.19 | 0.917 |  |
|  | Education (ref: less than high school) | High school diploma or equivalent | 2.05 | 1.46 | 2.90 | <0.001 |  |
|  |  | Some college | 1.87 | 1.34 | 2.61 | <0.001 |  |
|  |  | Bachelor or higher | 1.08 | 0.75 | 1.54 | 0.694 |  |
| **Use for Psychosis (e.g., paranoia, disorganized thinking, hearing voices that others can't** | | |  |  |  |  |  |
|  | Ethnicity (other vs white) | | 0.87 | 0.65 | 1.16 | 0.344 |  |
|  | Education (ref: less than high school) | High school diploma or equivalent | 1.91 | 1.09 | 3.33 | 0.024 |  |
|  |  | Some college | 1.85 | 1.07 | 3.18 | 0.027 |  |
|  |  | Bachelor or higher | 1.84 | 1.06 | 3.19 | 0.031 |  |
| **Use for Schizophrenia** | | |  |  |  |  |  |
|  | Ethnicity (other vs white) | | 1.06 | 0.77 | 1.46 | 0.700 |  |
|  | Education (ref: less than high school) | High school diploma or equivalent | 4.46 | 2.10 | 9.44 | <0.001 |  |
|  |  | Some college | 2.59 | 1.21 | 5.54 | 0.014 |  |
|  |  | Bachelor or higher | 1.95 | 0.89 | 4.26 | 0.093 |  |
| **Use for Drug or alcohol use** | | |  |  |  |  |  |
|  | Ethnicity (other vs white) | | 0.82 | 0.68 | 0.97 | 0.025 |  |
|  | Education (ref: less than high school) | High school diploma or equivalent | 1.46 | 1.10 | 1.96 | 0.010 |  |
|  |  | Some college | 1.38 | 1.03 | 1.84 | 0.029 |  |
|  |  | Bachelor or higher | 0.82 | 0.60 | 1.12 | 0.212 |  |
| †Use for each of the reasons were analysed in separate models; all models adjusted for gender, age, cannabis policy condition, ethnicity, and education; Education categories were: Less than high school; High school diploma or equivalent; Some college or technical/vocational training or certificate/diploma, or apprenticeship, or some university; and Bachelors degree or higher. | | | | | | | |

# S7 Proportion of International Cannabis Policy Study (ICPS) 2018 respondents by province or state of residencea (n=27,169)


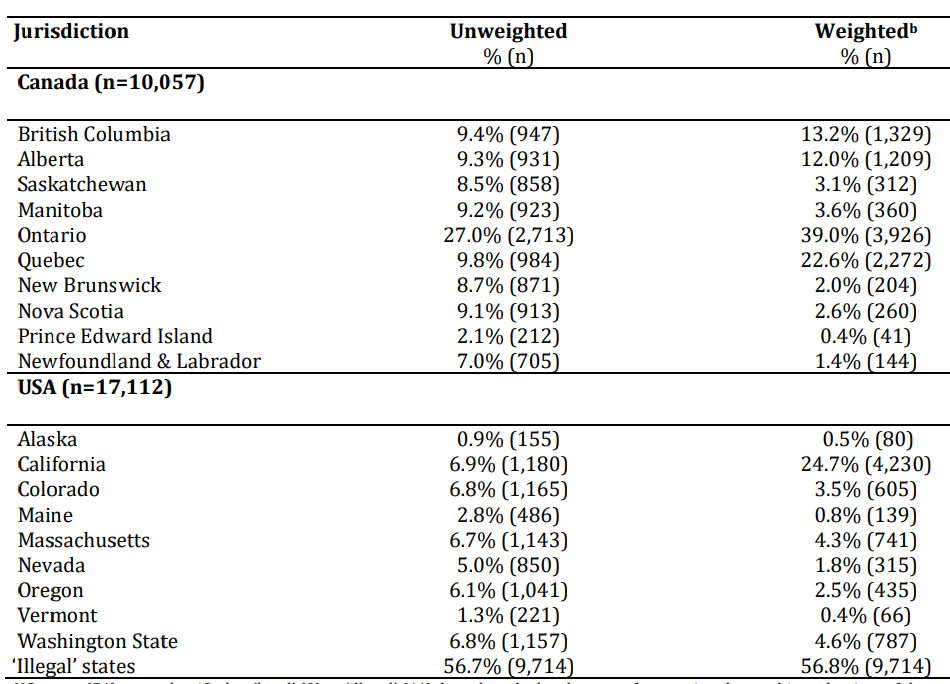


a. US states (51) were classified as ‘legal’ (9) or ‘illegal’ (41), based on the legal status of recreational cannabis at the time of the study (August 2018). The 9 US ‘legal’ states were oversampled compared to US ‘illegal’ states to ensure sufficient representation; Ontario was also oversampled.

b. Data are weighted to the national population, which are the inflation weights scaled back to the sample size of Canada, US legal states as a group, and US illegal states as a group.

Source: GOODMAN S, HAMMOND D. INTERNATIONAL CANNABIS POLICY STUDY TECHNICAL REPORT – WAVE 1 (2018). UNIVERSITY OF WATERLOO; WATERLOO, ON, CANADA. NOVEMBER 2019. AVAILABLE AT: http://cannabisproject.ca/methods/
